# Glutamate Provides Cytoprotective Effect for Astrocytes Against Ischemic Insult and Promotes Astrogliosis

**DOI:** 10.14336/AD.2023.0726

**Published:** 2024-02-01

**Authors:** Shao-Hua Yang, Yuanhong Sun, Raymond Berry, Gourav Roy Choudhury, Ali Winters, Kiran Chaudhari, Ran Liu

**Affiliations:** Department of Pharmacology and Neuroscience, University of North Texas Health Science Center, 3500 Camp Bowie Blvd, Fort Worth, Texas, 76107, USA

**Keywords:** ischemia, astrocytes, glutamate, energy metabolism, astrogliosis

## Abstract

Glutamate-mediated excitotoxicity has been extensively explored as a therapeutic target for the development of potential treatments of neurological disorders including stroke. However, the effect of glutamate on astrocytes under pathological conditions has been less studied. Using primary astrocyte culture, we determined the effect of glutamate on astrocytes against ischemic insult. Glutamate provided a cytoprotective effect and acted as an alternative substrate for ATP production in primary astrocytes against oxygen glucose deprivation reoxygenation insult, which was blocked by glutamate uptake inhibition. The cytoprotective effect of glutamate appears to be astrocyte-specific, as glutamate dose-dependently induces cytotoxic action in murine hippocampal HT-22 cell line. Interestingly, the cytoprotective effect of glutamate against glucose deprivation was short-last, as no protection was observed after 3-day glucose deprivation. We determined the metabolic phenotype of primary astrocyte cultured in glucose or glutamate. Primary astrocytes cultured in glutamate displayed a different metabolic phenotype when compared to those cultured in glucose, evidenced by higher basal and maximal oxygen consumption rate (OCR), higher ATP production and proton leak-coupled OCR, as well as lower glycolysis. Furthermore, glutamate exposure resulted in astrocyte activation, evidenced by an increase in astrocyte size and GFAP expression. Our study demonstrated that glutamate exerts a dual effect on astrocytes under ischemic condition. Glutamate provides an alternative substrate for energy metabolism in the absence of glucose, thereby protecting astrocytes against ischemic insults. On the other hand, glutamate exposure induces astrogliosis. Modulation of glutamate uptake and metabolism in astrocytes may provide novel targets for alleviating ischemic injury and improving function recovery after ischemic stroke.

## INTRODUCTION

Glutamate is one of the most abundant free amino acids and the principle excitatory neurotransmitter in the mammalian CNS [1]. Glutamate released from presynaptic glutamatergic neurons binds and activates both ionotropic and metabotropic glutamate receptors of postsynaptic neurons, playing a critical role in the function of the CNS [2]. Disruptions in glutamate homeostasis and excessive activation of ionotropic glutamate receptors excite neurons to death in a process referred to excitotoxicity, which has been well accepted as the primary mechanism of neuronal injury in various neuropathological conditions [3-5]. The extracellular glutamate concentration must be fine-tuned to avoid excitotoxicity, given the iniquitousness and abundancy of glutamate in the biological system.

Astrocytes are the major glial cells in the CNS and crucial to the brain function [6]. Astrocytes play critical roles in glutamate synthesis, reuptake, and disposal in the CNS [7, 8]. As a primary mechanism of neuronal injury in neurological disorders, glutamate-mediated excitotoxicity has been extensively explored as a therapeutic target for the development of potential treatments for neurological disorders including stroke [9]. Unfortunately, no anti-excitotoxicity treatment has been found to be effective for the treatment of any neurodegenerative diseases in clinical settings [4]. While astrocytes play a key role in maintaining glutamate homeostasis in the CNS, the effect of glutamate on astrocytes under pathological conditions has been rarely studied [8]. In the present study, we determined the effect of glutamate on primary astrocytes subjected to ischemic insult. We observed that glutamate exerted a dual effect on astrocytes under ischemic insult. While excessive glutamate exposure could provide a cytoprotective effect for astrocyte against oxygen glucose deprivation/reoxygenation by providing an alternative substrate for energy metabolism, it may also be involved in the induction of astrogliosis.

## MATERIALS AND METHODS

### Primary astrocyte cultures

All the experiments involving the use of animals were approved by The Institutional Animal Care and Use Committee (IACUC) of University of North Texas Health Science center (UNTHSC) at Fort Worth. Enriched primary cortical astrocyte cultures were prepared from postnatal C57BL6 pups as previously described [10]. Under aseptic conditions the brains were collected from 1-Day-old C57BL6 pups to obtain cortical tissue devoid of micro-vasculature. Cortical tissues were then digested with 0.25 % trypsin and triturated with Pasteur pipettes (3 different bore sizes) to prepare single cell suspension and plated in 10-cm cell culture plates. The cells were cultured at 37 °C with 5 % CO2 till preferred confluence was achieved. Microglia and oligodendrocytes were removed from the culture by constant shaking at 37 °C for 2 days with media changes every 24 hrs.

### In vitro oxygen glucose deprivation and glucose deprivation models

Primary astrocytes and HT-22 cells were subjected to oxygen glucose deprivation/reoxygenation (OGD/R) insult as previously described [10]. Briefly, astrocytes and HT-22 cells were cultured in a 96-well plate at a density of 3,000 / well in DMEM (Thermo Scientific) containing 10 % FBS and 1 % penicillin/streptomycin. On the day of experiment, the cells were washed twice with sterile phosphate buffer saline (PBS) and incubated in pyruvate and FBS free DMEM with glucose or glutamate for 6 hrs in an anaerobic chamber flushed with 95 % N2 (final oxygen concentration 0.1 %) and 5 % CO2 at 37 °C. During reoxygenation, the culture media was supplemented with D-glucose (11 mM) and pyruvate (1 mM) incubated in 5 % CO2, 95 % air at 37 °C for 24 hrs. For glucose deprivation insult, astrocytes seeded in 96-well plates were switched to pyruvate and FBS free DMEM with glucose or glutamate and cultured in normoxic condition for the indicated time (24 or 72hr). For glutamate uptake inhibition, astrocytes were pre-treated with TFB-TBOA (120 µM) for 2 hrs followed by treatment and OGD/R insult. For cell viability analysis, the culture media was replaced with 1 μM solution of Calcein AM and 5 μg/ml propidium iodide in PBS. The cells were then incubated at 37 °C for 5 minutes, and fluorescence was measured using a Tecan plate reader as described previously [11].

### Analysis of cellular ATP

Cellular ATP level was determined using an ATP assay kit (Invitrogen) as previously described [12]. Cells were detached with trypsin (Sigma) and washed twice with PBS and lysed with 100 µl of ATP assay buffer (500 mM Tricine buffer, pH 7.8, 100 mM MgSO4, 2 mM EDTA, and 2 mM sodium azide, 1 % Triton X-100). ATP reaction buffer (100 µl, 30 μg/ml D-luciferin, 20 μM DTT, and 25 μg/ml luciferase) was added to 10 µl cell lysate and the luminescence was measured using a Tecan Infinite M200 plate reader. The ATP values were determined from a standard curve and normalized to the protein concentration of each respective sample.

### Immunocytochemistry and Western blot

For immunofluorescence staining, primary astrocytes were cultured in cover slips in different conditions. After each treatment, cells were fixed in 4 % formaldehyde, permeabilized using 0.1 % Triton-X, and incubated overnight in primary antibody for glial fibrillary acidic protein (GFAP) and ALDH1L (Santa Cruz, 1:100, mouse) followed by species specific fluorescent secondary antibodies. Omitting primary antibodies were included for validation of the antibodies’ specificity and distinguishing the genuine target staining from background. Microscopic images were obtained using a ZEISS fluorescence microscope.

For Western blot analysis, primary astrocytes were seeded at a density of 3X10^5^ cells/well in 6-well plates in different culture medium for 24 hrs. RIPA lysis buffer (50 mM of Tris-HCl, pH 7.4, 1 mM EDTA, 150 mM NaCl, 1% Triton X 100 with phosphatase and protease inhibitors) was used for protein extraction. The protein concentrations of each sample were determined using Pierce 660 nm Protein Assay (Thermo Fisher Scientific, USA). The protein samples were resolved on SDS-PAGE (sodium dodecyl sulfate-polyacrylamide gel electrophoresis) and transferred to PVDF membrane. The membranes were blocked with 5% non-fat milk for 1 hr at room temperature. The membranes were probed with primary antibodies at 4 °C overnight (anti-ALDH1L1, 1:1000, Abcam, Cat #87117; anti-GFAP, 1:1000, Cell Signaling Technology, Cat #12389; anti-GAPDH, 1:5000, Santa Cruz Technology, Cat #sc-3223). The membranes were incubated with horseradish peroxidase-conjugated secondary antibodies (Jackson immunoResearch Laboratories, USA). Chemi-luminescence signal was detected with Biospectrum 500 UVP imaging system. FIJI-ImageJ (NIH, USA) was used to quantify the target protein bands’ density and normalized to GAPDH.

### Extracellular flux analysis

For metabolic analysis, primary astrocytes were seeded into Seahorse XFe 96 culture microplates at a density of 20,000 cells/well. One day prior to the experiment, the sensor cartridge was hydrated in deionized H2O in a non-CO2 incubator at 37 °C overnight. On the day of the experiment, deionized H2O was replaced with Seahorse calibrant in the sensor cartridge and placed in a non-CO2 incubator at 37 °C for 1 hr. The culture media was replaced with seahorse XF base medium containing 1 mM pyruvate, 2 mM glutamine, and 10 mM glucose in the XFe96 culture microplate and incubated in a non-CO2 incubator at 37 °C for 1 hr. Real-time metabolic analysis was conducted using Mito Stress Test kit (Agilent Technologies, CA). All measurements were normalized to the cell number using Hoechst staining.

### Statistical analysis

Results were expressed as mean ± standard error of mean (SEM), which is representative of at least 3 independent experiments with a minimum of 3 biological replicates. The Shapiro-Wilk test was used for normality test. Graph Pad Prism 5 was used for statistical analysis. When comparing two groups a t-test was used to identify any significant differences. For comparison of multiple groups, one-way analysis of variance was used and post-hoc Bonferroni test or the non-parametric Bonferroni test (for n<6) were conducted to identify the significant differences. A p-value of less than 0.05 was considered statistically significant.

**Figure 1.**
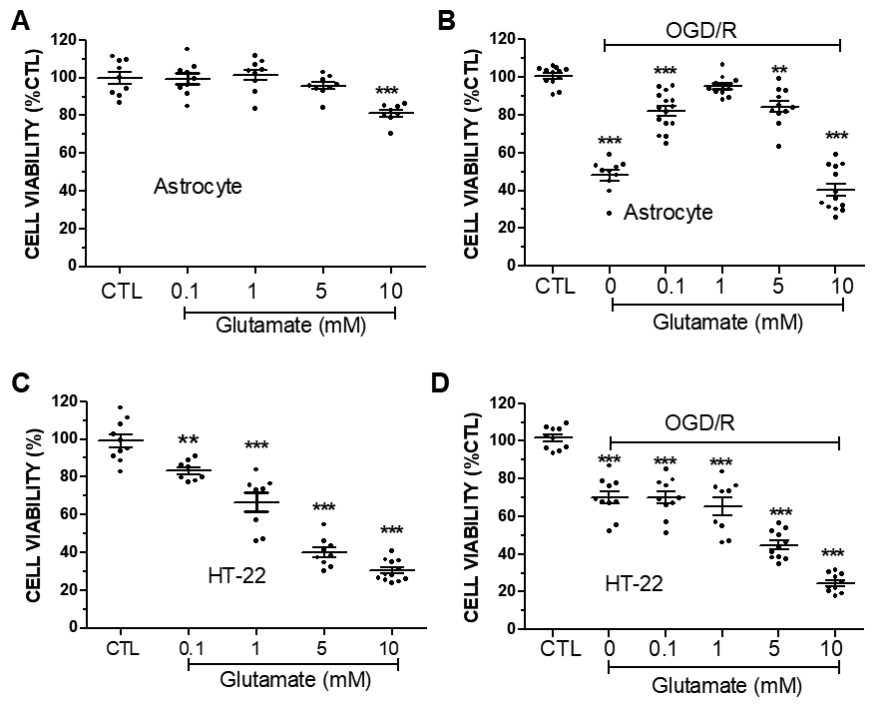
**Glutamate exposure rescued astrocytes from OGD/R induced cell death. (A)** The effect of glutamate exposure on cell viability in primary astrocytes under normoxia. **(B)** Concentration-dependent effect of glutamate exposure on astrocytes viability against OGD/R. **(C)** Glutamate concentration-dependently induced cytotoxicity in HT-22 cells under normoxia. **(D)** Glutamate exaggerated glutamate toxicity in HT-22 cells subjected to OGD/R insult. n=8-15 each group, ** p<0.01, *** p<0.001 vs control (CTL).

**Figure 2.**
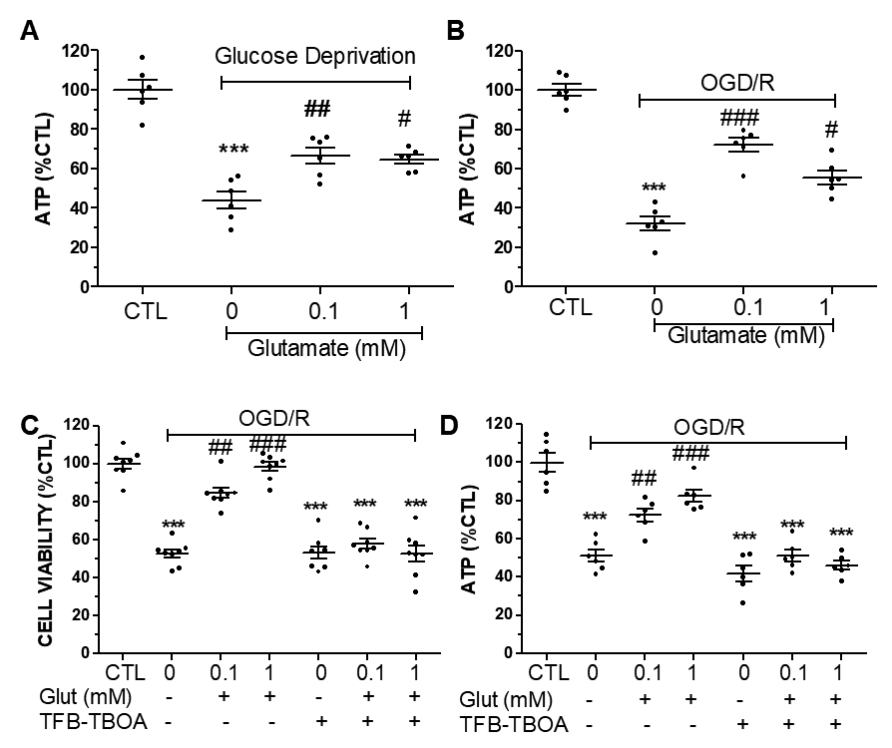
**Effect of glutamate exposure on ATP levels in primary astrocytes subjected to glucose deprivation or OGD/R insult. (A)** Glutamate exposure significantly increased ATP level in primary astrocytes as compared to control (CTL) in glucose deprivation situation. **(B)** Glutamate exposure significantly increased ATP level in primary astrocytes as compared to control in OGD/R insult. **(C)** Quantitative cell viability analysis demonstrated that TFB-TBOA blocked the cytoprotective effect of glutamate in primary astrocytes. **(D)** ATP assay demonstrated that glutamate exposure increased ATP production in primary astrocytes, which was blocked by TFB-TBOA. n=6-8 each group, *** p< 0.001 vs CTL, # p<0.05, ## p<0.01, ### p<0.001 vs 0 glutmate.

## RESULTS

### Glutamate protects astrocytes against short-term oxygen-glucose deprivation/reoxygenation (OGD/R) insult

We evaluated the effect of glutamate on cell viability of primary astrocytes under normoxic conditions. Exposure of glutamate for 24-hr had no cytotoxic effect on astrocytes until reaching 10 mM concentration (Fig. 1A). Primary astrocytes were exposed to 6-hr OGD followed by 24-hr reoxygenation, which resulted in ~50% cell death. Glutamate exposure at the concentrations of 0.1 and 1 mM significantly attenuated astrocyte death induced by OGD/R insult (Fig. 1B). The cytoprotective effect of glutamate disappeared at concentrations of 10 mM. We further determined if the cytoprotective action of glutamate against OGD/R insult was astrocyte-specific. Glutamate induced a dose-dependent cell death in HT-22 cells, a murine hippocampal cell line. When superimposed to OGD/R insult, glutamate did not provide any cytoprotection at the concentrations of 0.1 and 1 mM, but increased cell death at the concentrations of 5 and 10 mM (Fig. 1C & D).

**Figure 3.**
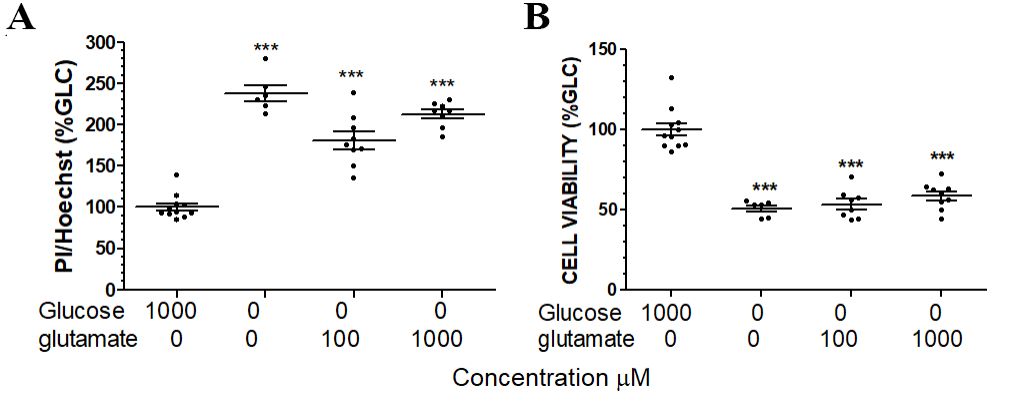
**Glutamate has no cytoprotective effect against 72-hr glucose deprivation. (A)** Propidium iodide assay depicts a significant increase cell death after 72-hr glucose deprivation and no protection of glutamate at 100 and 1000 μM. **(B)** Calcium AM viability assay depicts a significant decrease cell viability after 72-hr glucose deprivation and no pro-tection of glutamate at 100 and 1000 μM. n=6-11 each group, *** p<0.001 vs glucose 1000 µM/glutamate 0 µM.

We determined the effect of glutamate on cellular energetics under both glucose deprivation and OGD/R. Glucose deprivation significantly decreased ATP production in primary astrocytes. Glutamate (0.1 and 1 mM) significantly increased ATP production as compared with glucose deprivation controls (Fig. 2A), suggesting that glutamate could sustain astrocytes ATP production in the absence of glucose. Similarly, OGD/R dramatically decreased ATP production in primary astrocytes, which was attenuated by the exposure of 0.1 and 1 mM glutamate (Fig. 2B). These data indicated that glutamate could provide an alternative substrate for ATP production in the absence of glucose and/or oxygen. To explore whether glutamate uptake inhibition alleviates the cytoprotective effect of glutamate against OGD/R insult, primary astrocytes were pre-treated with TFB-TBOA, a glutamate uptake inhibitor, then subject to OGD/R. TFB-TBOA pretreatment completely blocked the cytoprotective effect of glutamate against ischemic insult (Fig. 2C). Consistently, TFB-TBOA pretreatment completely blocked the increase of ATP production in glutamate-treated astrocytes (Fig. 2D). We further determined the effect of glutamate on 72-hr glucose deprivation. No protection upon glutamate treatment was observed by calcium AM and propidium iodide assay (Fig. 3).

### Glutamate sustains a different metabolic phenotype in astrocytes compared to glucose

**Figure 4.**
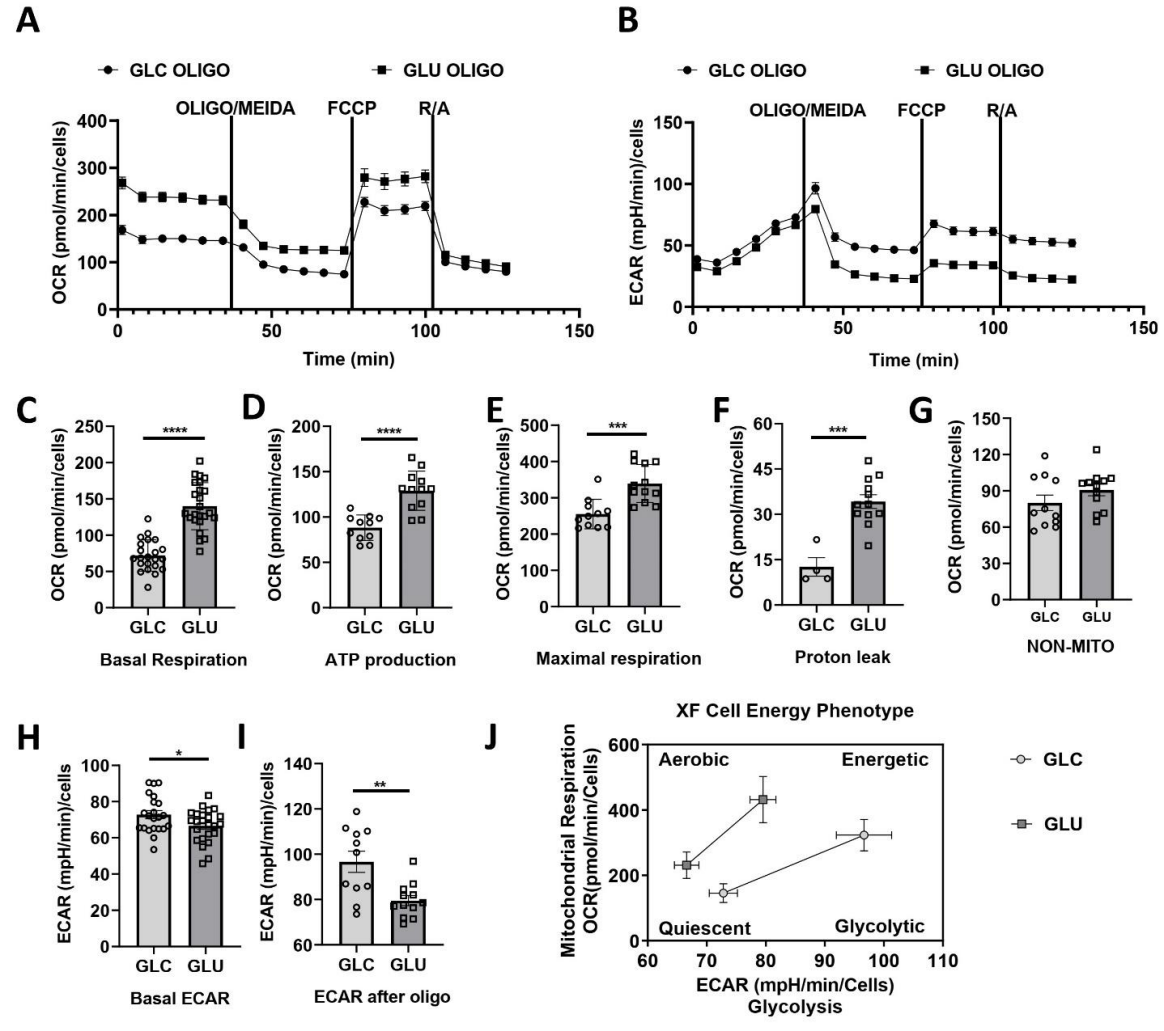
**Glutamate sustains astrocytes with different metabolic phenotype from glucose. (A, B)** Continuous tracing of oxygen consumption rate (OCR) and extracellular acidification rate (ECAR) of primary astrocytes cultured in glucose (Glc) or glutamate (Glu) before and after inhibition of mitochondrial complex V (oligomycin, oligo), addition of mitochondrial oxidative phosphorylation uncoupler FCCP, and inhibition of mitochondrial complex I/III (rotenone/antimycin A) in the same 96-welled plate. **(C to G)** Scatter dot plots depict basal, ATP production-coupled, proton leak-coupled, non-mitochondrial OCR, and maximal respiration of primary astrocytes cultured in 1 mM glucose or 100 μM glutamate. **(H- I)** Scatter dot plots depict basal and stressed ECAR of primary astrocytes cultured in glucose or glutamate. **(J)** Metabolic profile of primary astrocytes cultured in glucose or glutamate. n=4-12, *** p<0.001.

We determined the metabolic phenotype of primary astrocytes cultured in glutamate or glucose using Seahorse XFe Mito Stress Test. Compared with astrocytes cultured with glucose (1 mM), primary astrocytes with 90-min pre-incubation of glutamate have significantly higher basal OCR, ATP production-coupled OCR, maximal OCR, and proton leak-linked OCR (Fig. 4A, C, D, E, F, and G). In addition, glutamate-treated (100 μM) primary astrocytes display significantly lower basal and stressed ECAR as compared with glucose-cultured astrocytes (Fig. 4B and H). Overall, glutamate treatment switched astrocytes from glycolytic to aerobic phenotype (Fig. 4J).

### Glutamate induces astrocyte activation

**Figure 5.**
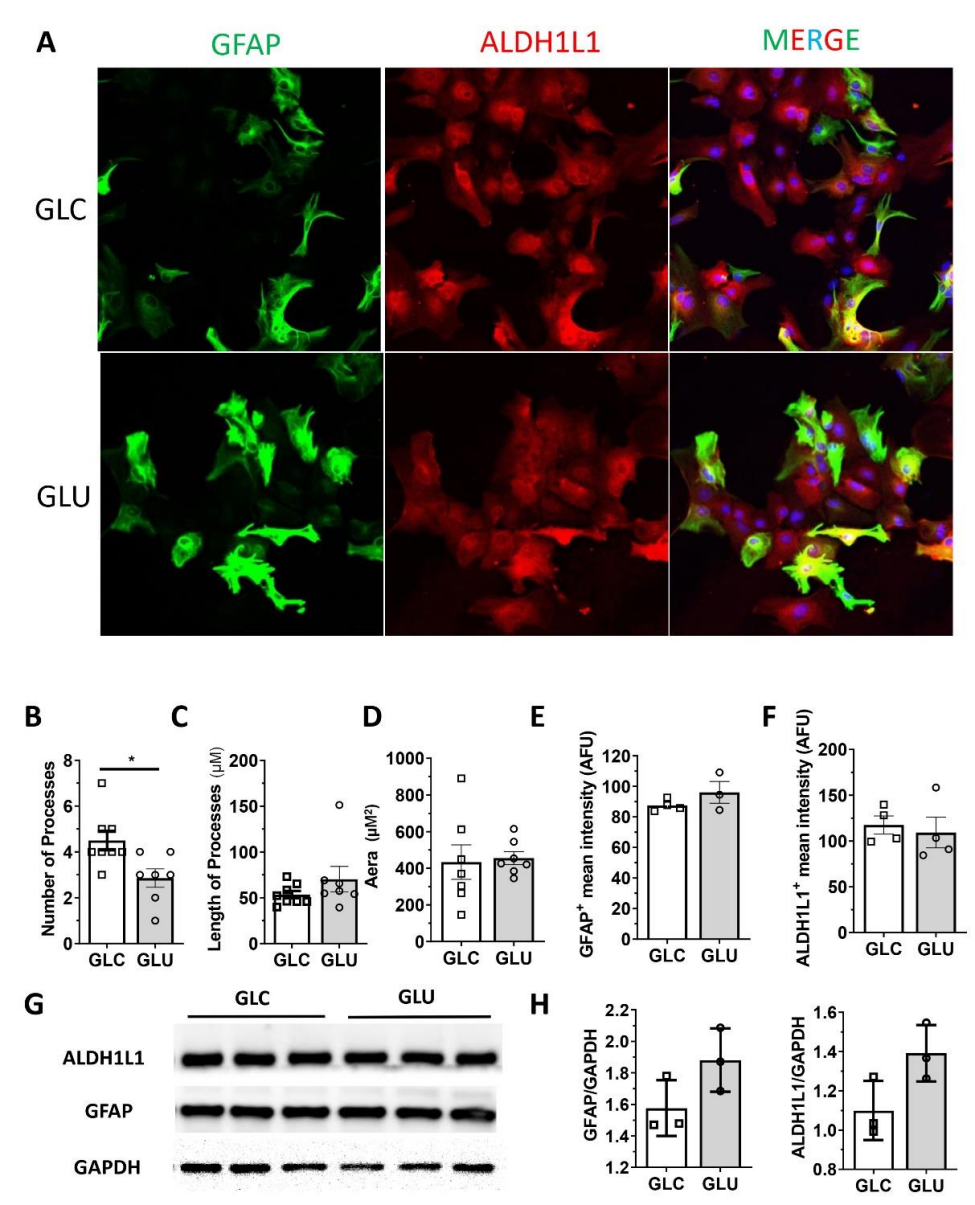
**Glutamate exposure induced astrogliosis. (A)** Representative fluorescent microscopic images of immunocytochemistry of GFAP and ALDH1L in primary astrocytes cultured in glucose (1 mM) or glutamate (0.1 mM) for 24-hr. **(B-F)** Quantitative analysis of number of processes, process length, astrocyte size, GFAP intensive, and ALDH1L1 intensity of primary astrocytes cultured in glucose or glutamate. **(G, H)** Representative Western blots and quantitative analysis of ALDH1L, GFAP, and GAPDH expression in primary astro-cytes cultured in glucose or glutamate. n=3-7, * p<0.05.

We determined the effect of glutamate on astrocyte activation. Immunocytochemistry demonstrated that 1 mM glutamate significantly increased GFAP expression under normoxic condition (Fig. 5A-F). Similarly, Western blot analysis indicated primary astrocytes cultured in 1 mM glutamate display a strong trend of increased GFAP expression as compared with glucose-cultured primary astrocytes (Fig. 5G and H).

## DISCUSSION

Glutamate is a key metabolite, and principle excitatory neurotransmitter in the CNS [13]. Glutamate homeostasis in the brain is maintained by its well-orchestrated synthesis, release, reuptake, and metabolism [14]. As the blood-brain barrier essentially prevents the entry of glutamate into the CNS, most of the glutamate in the brain is synthesized de novo in astrocytes, which requires both pyruvate carboxylase and glutamine synthetase [7]. In the CNS, excitatory amino acid transporters (EAATs) remove glutamate from extrasynaptic sites and synaptic cleft. Although EAATs are expressed in almost all cell types in the CNS including neurons, metabolically handicapped neurons rely on astrocytes for clearance of extracellular glutamate [15]. Anatomically, astrocytic processes completely enclose glutamatergic synapses, which not only prevent glutamate from diffusing from the synaptic cleft but also enable quick glutamate uptake. The tripartite synaptic compartmentation results in a steady traffic of glutamate from neurons to astrocytes and back to neurons via glutamate-glutamine cycle, enabling glutamatergic neurons to function efectively with exceptional signal-to-noise ratios and avoid excitotoxicity [16, 17].

Under physiological condition, extracellular glutamate concentrations maintain at 15 to 30 µM in the brain [18]. At the glutamatergic synapses, neurons and astrocytes are exposed to higher concentrations of glutamate of a few mM that last less than 1 milli-second [19]. Under ischemia, the affected brain tissue could endure more than 100-fold increase of extracellular glutamate concentrations for a much longer duration [18]. In neurons, glutamate binds to NMDA receptors, which are highly permeable to Ca2+ and distributed widely on CNS neurons, hence initiates excitotoxicity. On the other hand, astrocytes are generally resistant to excitotoxic insult and the effect of prolonged exposure of high concentrations of glutamate on astrocyte viability has been less clear [20]. In the present study, we determined the effect of glutamate on astrocytes. Under normoxia with glucose, glutamate did not induce astrocyte death until 10 mM concentration, which may not be reached after cerebral ischemia in vivo. Interestingly, glutamate provided a cytoprotective effect against OGD reoxygenation in primary astrocytes at the concentrations of 0.1 and 1 mM. Furthermore, the cytoprotective action of glutamate against ischemic insult appears astrocyte-specific, as no protective effect of glutamate was observed in neuronal HT-22 cell line subject to ischemic insult.

**Figure 6.**
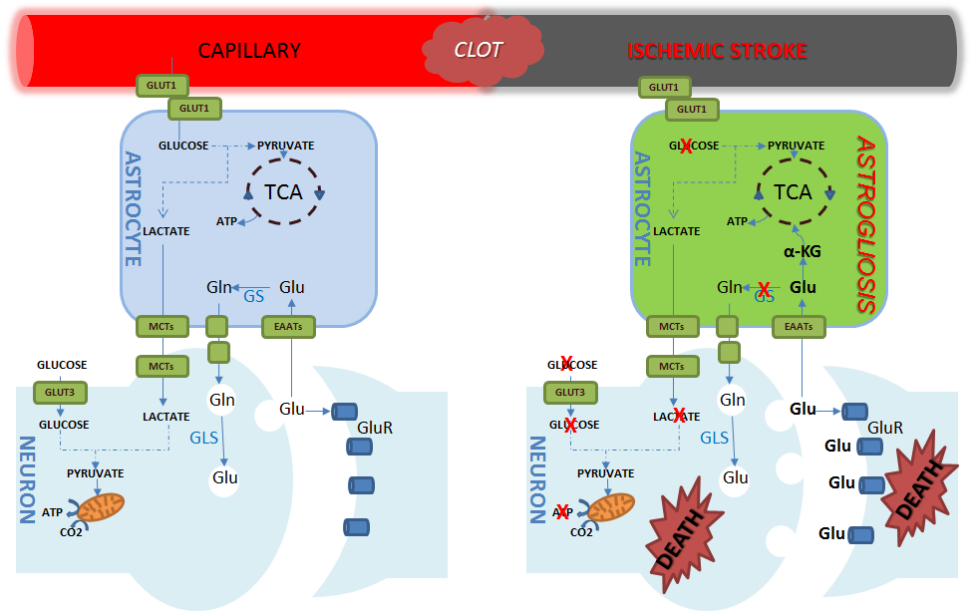
**Schematic representation of glutamate metabolism under normal and ischemic condition**. In physiological condition, neurons released glutamate (Glu) is up taken by astrocyte through excitatory amino acid transporters (EAATs), converted into glutamine by astrocyte-specific glutamine synthetase (GS) to be released and taken up by neighbor neurons. In neurons, glutamine (Gln) is converted back to glutamate by glutaminases (GLS). Under ischemic stroke, extensive glutamate released from neurons results in intracellular accumulation of glutamate in astrocytes. In astrocyte, glutamate is converted into α-ketoglutarate (α-KG) which enables the truncated TCA cycle and maintenance of ATP production, providing cytoprotective effect for astrocyte against ischemia and inducing astrogliosis. GLUT1, glucose transporter 1; GLUT3, glucose transporter 3; MCTs, mono-carboxylate transporters.

The mammalian brain depends on glucose as its main source for energy metabolism [21]. Astrocytes have a unique energy metabolism profile as compared to neurons. Besides being the principle excitatory neurotransmitter, glutamate functions as an important metabolic substrate, which is oxidized to produce ATP in the brain *in vivo* and in cultured astrocytes [7, 22, 23, 24]. Consistently, exposure of glutamate at 0.1 and 1 mM significantly increased ATP production in primary astrocytes in the absence of glucose and/or oxygen. We further explored whether glutamate uptake inhibitor could alleviate the cytoprotective and bioenergetic effects of glutamate in astrocytes against ischemic insult. Astrocytes were pre-treated with TFB-TBOA, a glutamate uptake inhibitor [25], and then subjected to OGD/R. TFB-TBOA pre-treatment completely blocked the cytoprotective and energy boosting effects of glutamate in astrocytes subjected to ischemic insult.

Several important aspects have been found that may contribute to the lower vulnerability of astrocytes to ischemic insult than neurons [26]. First, astrocytes have a lower density of ionotropic glutamate receptors than neurons and have better antioxidant capacity [26]. Second, astrocytes store energy in the form of glycogen which might offer protection against glucose starvation [27]. Third, astrocytes rely more on glycolytic metabolism and hence are less susceptible to hypoxia, which enables them to maintain ATP level longer than neurons during ischemia [28, 29]. We expected that the unique glutamate metabolism provides a critical role that underlies the selective destruction of neurons over astrocytes during ischemia. Uptaken glutamate may follow at least two metabolic fates in astrocytes. Glutamate is converted into glutamine by astrocyte-specific glutamine synthetase to be released and taken up by neighbor neurons via glutamate-glutamine cycle [30]. In addition, glutamate can be converted into α-ketoglutarate which enables the truncated tricarboxylic acid (TCA) cycle and ATP production [31]. Prolonged application of glutamate has been shown to switch off the astrocytic metabolism from glycolytic to oxidative, manifested as a stimulation of mitochondrial activity, decreased glucose uptake and decreased glycolytic lactate production [32]. Consistently, it has been shown that increased influx of glutamate into astrocytes favors its flux to the TCA cycle instead of the glutamate-glutamine cycle [33]. We predicted that glutamate could be used as an alternative substrate for energy metabolism in the absence of glucose, thus providing a cytoprotective effect for astrocytes against ischemic insult. Indeed, our extracellular flux analysis indicated that glutamate sustains astrocytes even higher basal, ATP production-linked, and proton-linked OCR, and maximal respiration. Interestingly, glutamate seems to only provide cytoprotection against short-term OGD or glucose deprivation as no such protection was observed in primary astrocytes under 72-hr glucose deprivation.

Astrocytes respond to all forms of CNS insult by a process commonly referred to as reactive astrogliosis characterized as hypertrophy of cellular processes and upregulation of intermediated filament proteins as glial fibrillary acidic protein (GFAP) [34-36]. Increasing research findings have suggested that reactive astrogliosis may exert both beneficial and detrimental effects in a context-dependent manner regulated by specific molecular signaling cascades [37-40]. Perfusion of glutamate *in vivo* has been shown to induce reactive astrogliosis, accompanied by a persistent cytosolic accumulation of glutamate, and selective decrease of glutamine synthetase activity [41]. We observed that glutamate exposure increased GFAP expression in primary astrocytes. Reactive astrocytes undergo not only morphological, molecular, functional but also metabolic remodeling in response to CNS damage [42]. The astrocytes’ glycolytic metabolism is important for the brain function potentially through providing lactate to support neuron energy metabolism [31]. Our Sea-horse flux analysis demonstrated that glutamate exposure switches astrocyte from a glycolytic energy phenotype to aerobic phenotype, thus, may provide less biosynthetic support for neurons.

In summary, the present study demonstrated that exposure of high concentrations of glutamate may have a dual effect on astrocytes after ischemic stroke. On one hand, high concentrations of glutamate may provide an alternative substrate for energy metabolism and cytoprotective effect against ischemic insult. On the other hand, high concentrations of glutamate may lead to cytosolic accumulation of glutamate and induce astrogliosis after ischemic stroke (Fig. 6). Our study has provided important insight regarding the role of glutamate in pathophysiology of astrocytes during ischemic stroke. In addition to excitotoxicity, modulation of glutamate uptake and metabolism in astrocytes may provide novel targets for alleviating ischemic injury and improving function recovery after is-chemic stroke.
